# Decoding surgical proficiency and complexity: a machine learning framework for robotic herniorrhaphy

**DOI:** 10.1007/s00464-025-12412-x

**Published:** 2025-12-02

**Authors:** Thomas H. Shin, Abeselom Fanta, Fahri Gokcal, Mallory Shields, Cigdem Benlice, O. Yusef Kudsi

**Affiliations:** 1 Division of General Surgery, Department of Surgery, University of Virginia School of Medicine, 1300 Jefferson Park Avenue, Charlottesville, VA 22903 USA; 2 Division of General and Gastrointestinal Surgery, Brigham and Women’s Hospital, Harvard Medical School, Boston, MA USA; 3 Intuitive Surgical, Sunnyvale, CA USA; 4 Department of General Surgery, School of Medicine, Acibadem Mehmet Ali Aydinlar University, Istanbul, Turkey; 5 Department of General Surgery, School of Medicine, Istanbul Health and Technology University, Istanbul, Turkey

**Keywords:** Objective performance indicators, Machine learning, Robotic ventral hernia repair

## Abstract

**Objective:**

To evaluate the predictive value of objective performance indicators (OPIs) for case complexity assessment and explore their role in quantifying skill acquisition during robotic ventral herniorrhaphy.

**Summary background data:**

Despite advances in herniorrhaphy techniques, unclear metrics of case complexity have significant implications for operative planning, resource allocation, and patient outcomes. While existing complexity definitions rely primarily on clinical factors external to operator behavior, the expanding adoption of robotic platforms in ventral hernia repair provides unprecedented access to quantifiable surgical performance metrics. However, the relationship between these objective performance indicators and both case complexity and skill development remains incompletely understood, representing a gap that machine learning approaches may help address.

**Methods:**

OPI and clinical data from 561 consecutive robotic ventral hernia repairs over eight years were analyzed using iterative ensemble machine learning models to predict case complexity. Dimensional reduction analyses using t-distributed stochastic neighbor embedding tracked skill evolution, with Euclidean distances calculated between successive cases to quantify skill acquisition over time.

**Results:**

Gradient boosting models integrating clinical and OPI variables achieved F1 score of 0.87, while OPIs alone scored 0.58. Longitudinal analysis revealed high OPI variability during early cases, stabilizing within 10 months despite increasing case complexity, indicating skill acquisition may compensate for procedural difficulty. Dimensional reduction analyses captured this evolution through weighted Euclidean distances.

**Conclusions:**

Objective performance indicators poorly predict case complexity independently, yet their temporal evolution reveals surgical skill acquisition. The concurrent stabilization of OPI stochasticity and progression to more complex cases demonstrates that surgical proficiency and complexity assessment are interdependent phenomena, establishing digital metrics as tools for understanding the dynamic relationship between surgeon learning and case difficulty.

**Supplementary Information:**

The online version contains supplementary material available at 10.1007/s00464-025-12412-x.

Despite advances in herniorrhaphy technique and approaches in recent decades, unclear metrics of case complexity has significant implications in patient care and outcomes, ranging from operative planning, approach of repair, resource allocation, concordance with operator skill level, and perioperative patient risk stratification [1, 2]. While existing definitions of hernia complexity exist, these mostly rely on perioperative patient and hernia characteristics exogenous of operator factors, rendering their predictive power of postoperative outcomes incomplete. Objective characterization of hernia complexity can lead towards a new practice paradigm where herniorrhaphy technique and perioperative planning can be further standardized and enable outcomes optimization [3].

The expanding adoption and translation of complex abdominal wall reconstructions in minimally invasive surgery has led to a surge in robotic-assisted laparoscopic ventral hernia repairs. By leveraging the robotic platform, continued innovations within the field has led to an uptake of laparoscopic complex abdominal wall reconstruction by leveraging the robotic platform [4–7]. The increasing integration of robotic surgical platforms in hernia repairs provides unprecedented access to quantifiable metrics of surgical performance through objective performance indicators (OPIs) [8, 9].

While the relationship between OPIs and surgical outcomes has been well documented across surgical specialties, their utility in quantifying case complexity and technical skill development in general surgery, particularly in hernia repair, remains incompletely understood [10–27]. The digital nature of robotic platform-derived metrics presents a unique opportunity to employ machine learning algorithms, enabling AI-driven analyses to discover patterns and relationships without relying on predetermined models or assumptions [25–27]. In this study, we leverage robotic platform-derived OPI data from ventral hernia repairs to elucidate whether objective measures of surgical behavior can capture technical skill acquisition and case complexity. Through this analysis, we aim to establish a framework for understanding how digital surgical metrics can inform our assessment of technical proficiency and operative difficulty.

## Materials and methods

### Patient selection

Data from 561 consecutive robotic-assisted ventral hernia repairs were prospectively obtained and retrospectively reviewed. All operations were performed by a single surgeon with surgical trainees (clinical fellows) at a high-volume academic medical center between February 2013 and November 2022. All operations were completed with the da Vinci® robotic system (Intuitive Surgical, Sunnyvale, CA). Cases with concurrent procedures outside of ventral hernia repair were excluded, resulting in 468 cases in total.

### Feature engineering and machine learning models predicting case complexity

In the data preprocessing stage for OPI computation, surgeon-to-console mapping was conducted based on the robotic data stream and recurring console usage. Dual-console cases with only one active console were categorized as single-console cases. Retrospective review of records confirmed single-console cases were indeed with only one surgeon for the entirety of the case. To address the ordinality in variables such as hernia positions, etiology, location, and concomitancy, one hot encoding was employed. This encoding method was also applied to the attending and non-attending surgeons to eliminate experience bias. Additionally, the number of previous RVHR cases performed by each surgeon was used to represent experience level.

OPIs and patient factors were incorporated into iterative ensemble machine learning models to predict case complexity using a supervised methodology as previously described [11]. OPIs and operative metrics were prospectively collected from robotic data streams and represented as total event counts, durations, rates, and normalized durations (total event count divided by case duration).

Primary endpoint was prediction of case complexity as defined by comprehensive patient and hernia characteristics. Hernia complexity was determined using criteria established through consensus agreement and systematic literature review by an international collaboration of expert hernia surgeons. These criteria incorporated hernia size, soft tissue integrity, patient comorbidities, and the clinical scenario surrounding hernia repair [2]. Various ranges of model parameters were selected to perform model iteration. Iterative machine learning models utilized to classify complexity include the following: Categorical Boosting (CatBoost), Random Forest, Light Gradient Boosting Machine, Extreme Gradient Boosting, Decision Tree, Adaptive Boosting, Support Vector, and k-nearest neighbor models. Conventional logistic regression models were also employed as comparison. Variants from each model with the highest F1 score (harmonic mean between precision and recall) were selected as the representative model for each outcome measure. We employed fivefold cross-validation to demonstrate model robustness and used shapley additive explanations values (SHAP) to determine ranked feature importance in final selected model.

### Dimensional reduction for learning curve analysis

For the learning curve analysis, cases were binned by the month of operation. A high-dimensional matrix was employed to represent number of cases, OPIs, and complexity for each given month. To facilitate dimensional reduction, t-distributed stochastic neighbor embedding (t-SNE) was used. Multiple-case components related to the same OPI for a specific month were averaged. The Euclidean distance between consecutive bins was calculated to measure similarities between neighboring time clusters. To account for bins with no reported cases and variable case volumes, the distances were weighted by number of days between consecutive bins and volume of cases. Savitzky–Golay smoothing filter with 5-moving window was applied to smooth the distance data [28]. The mathematical expression is demonstrated in Eqs. 1 and 2.

Given time bin t, the components $i$ and $j$ of cluster p corresponding to one OPI and Euclidean distance d are computed as follows:

1 $${p}_{i,j|t}=tSNE(\left[cases,OPIs,complexity\right])$$

2 $${d}_{t,t+1}=\sqrt{\frac{{w}_{t+1,t}}{{c}_{t+1}}{\sum }_{n\in (i,j)}{\left({p}_{n|t+1}-{p}_{n|t}\right)}^{2}},$$

where $w_{t + 1,t} = last day_{t + 1} - last day_{t}$ and $c_{t + 1}$ is number of cases at bin $t + 1$.

Exponential decay functions were fitted to the eigenvalues obtained from eigendecomposition, and the convex hull area was applied to the low-dimensional components of each bin to estimate the temporal clustering of OPI, representing evolution of skill acquisition. This was used to segment successive cases as “novice” versus “expert based on the expected values derived from the curve fitting as follows:

$$f_{i} \left( x \right) > E\left[ {f_{i} \left( x \right)} \right] \to Novice$$

$$f_{i} \left( x \right) \le E\left[ {f_{i} \left( x \right)} \right] \to Expert$$

For $i \in \left( {eigendecomposition,convex hull} \right)$ With this segmentation, the previous ensemble machine learning models were re-run as described in the aforementioned subsection to determine whether OPIs can function as independent predictors of case complexity while accounting for surgeon skill level.

### Regulatory oversight and ethics

This study was completed under Institutional Review Board approval with waiver of informed consent (Good Samaritan Medical Center IRB protocol HW11-22). Funding support for this research was obtained through an Intuitive Surgical Foundation Research Grant (Sunnyvale, CA).

## Results

### Patient and hernia characteristics

Patient characteristics and perioperative comorbidities in study cohort are summarized in Table 1. Median age of patients was 55 years and 49.1% were female with a median body mass index of 31.9 kg/m^2^. Over half of patients in our study had at least one comorbidity, with hypertension being the most prevalent (54.5%) followed by previous smoking history (23.5%) and diabetes mellitus (19.7%). Emergent cases comprised 5.6% (29 cases) of all operations. Notably, the median American Society of Anesthesiology Physical Status Classification score was 2 with an interquartile range of 2–3, suggesting the median patient in our serious had mild to moderate systemic disease controlled medically at baseline.

**Table 1:** Patient characteristics and perioperative comorbidities

	Cases (n = 468)
Age, median years (IQR)	55 (44–66)
Female sex	230 (49.1)
Body mass index, kg/m^2^ (IQR)	31.9 (28.2–36.6)
ASA Physical Status Classification, median (IQR)	2 (2–3)
ASA 1	25 (5.3)
ASA 2	213 (45.5)
ASA 3	225 (48.1)
ASA 4	45 (1.1)
Patient comorbidities	
Hypertension	255 (54.5)
Previous myocardial infarction	9 (1.9)
Coronary artery disease	37 (1.9)
Chronic obstructive pulmonary disease	53 (11.3)
Smoking history	110 (23.5)
Diabetes mellitus	92 (19.7)
Immunosuppression use	6 (1.3)
History of previous wound infections	40 (8.5)
Steroid use	32 (6.8)
Hypoalbuminemia	6 (1.3)

Incidence denoted with percent unless otherwise noted

*IQR* interquartile range, *ASA* American Society of Anesthesiology

Hernia characteristics are outlined in Table 2. Based on expert consensus definitions of hernia complexity as outlined by Slater et al., the study cohort comprised of 38.7% minor, 45.5% moderate, and 15.8% major complexity hernias. This correlates with other metrics of hernia grading systems, including the Hernia-Patient-Wound classification (HPW) and Modified Ventral Hernia Working Group (VHWG) grading system, which found most hernias to be HPW stage 2 (73.9%) and VHWG grade 2 (78.6%). Hernia repair approach was varied: robotic intraperitoneal onlay 29.3%, robotic transabdominal preperitoneal 23.5%, robotic Rives-Stoppa 27.4%, and robotic transversus abdominis release 19.9%.

**Table 2:** Hernia characteristics and intraoperative factors

	Cases (n = 468)
Hernia complexity class	
Minor	181 (38.7)
Moderate	213 (45.5)
Major	74 (15.8)
Hernia etiology	
Primary ventral	248 (53)
Incisional	213 (45.5)
Both	7 (1.5)
Hernia location	
Midline	432 (92.3)
Non-midline	21 (4.5)
Both	15 (3.2)
Multiple hernia defects	71 (15.2)
Hernia-Patient-Wound classification	
Stage 1	90 (19.2)
Stage 2	346 (73.9)
Stage 3	31 (6.6)
Stage 4	1 (0.2)
Modified Ventral Hernia Working Group grade	
Grade 1	92 (19.7)
Grade 2	368 (78.6)
Grade 3	8 (1.7)
Emergent or urgent repair	26 (5.6)
Mesh/hernia repair approach	
rIPOM	137 (29.3)
rTAPP	110 (23.5)
rRS	128 (27.4)
rTAR	92 (19.9)
Hernia defect characteristics	
Area, median cm^2^ (IQR)	12.6 (3.14–25.1)
Length, median cm (IQR)	4 (2–7)
Width, median cm (IQR)	4 (2–5)
Mesh characteristics	
Area, median cm^2^ (IQR)	225 (144–300)
Length, median cm (IQR)	15 (12–20)
Width, median cm (IQR)	15 (12–15)
Mesh-to-hernia defect ratio, median (IQR)	15 (9–21.6)
Console time, median minutes (IQR)	60 (40.0–113.5)
Skin-to-skin operative time, median minutes (IQR)	80 (53–132)
Estimated blood loss, mL (IQR)	5 (5–10)
Drain placement	6 (1.3)
Intraoperative complications	10 (2.1)
Robotic platform used	
daVinci Si	248 (53)
daVinci Xi	220 (47)

Incidence denoted with percent unless otherwise noted

*IQR* interquartile range, *rIPOM* robotic intraperitoneal onlay mesh, *rTAPP* robotic transabdominal preperitoneal, *rRS* robotic Rives-Stoppa, *rTAR* robotic transversus abdominis release

### Iterative ensemble machine learning models on case complexity

**Fig. 1:**
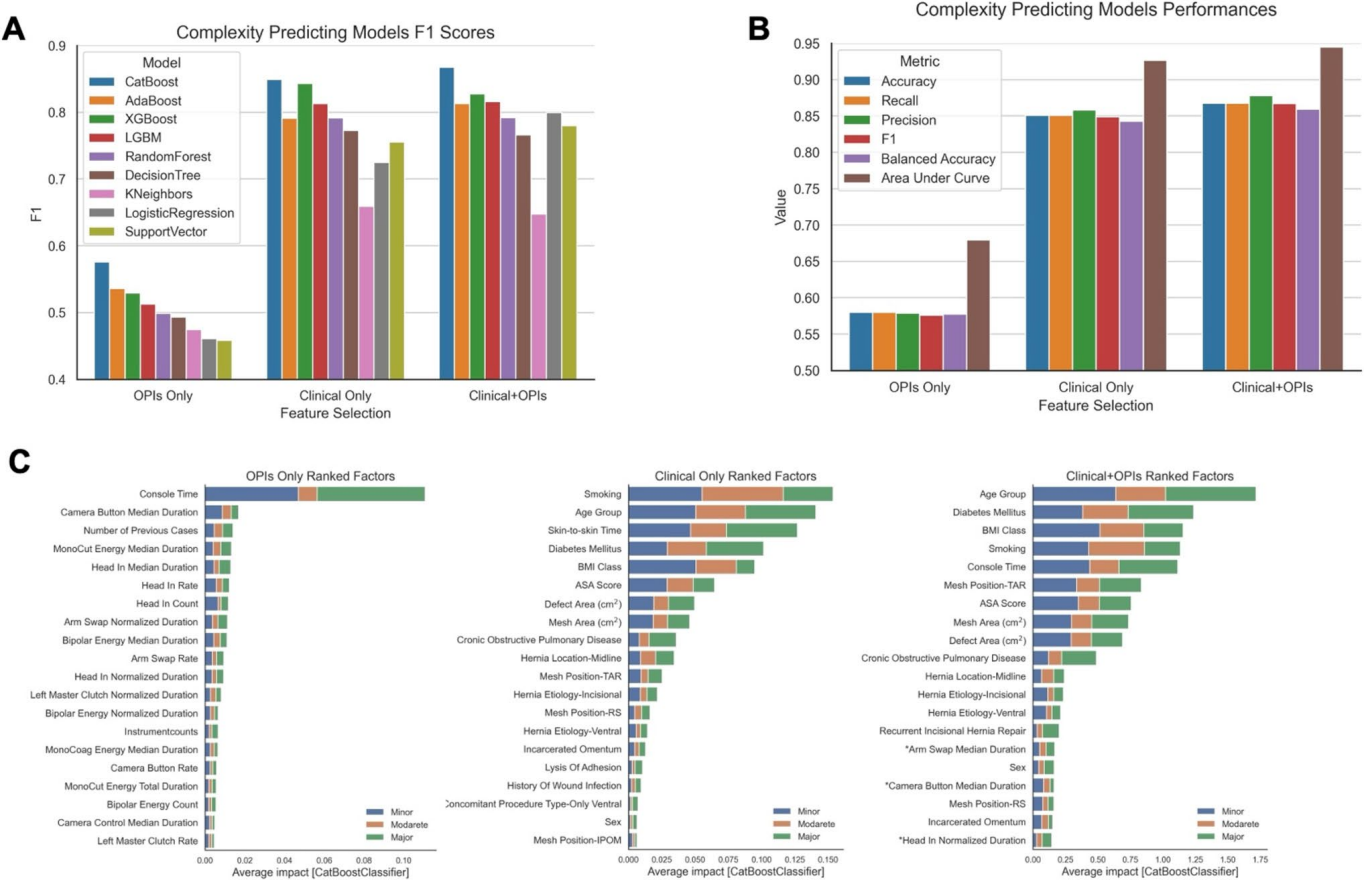
Iterative ensemble machine learning models predicting case complexity using clinical and OPI factors. **A** Summary of F1 scores of each iterative machine learning model using OPI factors only, clinical factors only, and clinical and OPI factors together. **B** Performance metrics of the best performing complexity predictive models using OPI factors only, clinical factors only, and clinical and OPI factors combined. **C** SHAP values of top ranked factors in each model, segmented by prediction for minor, moderate, and major complexity cases

Multiple iterations of logistic regression, k-nearest neighbors, and ensemble models were used to identify CatBoost as the best performing model predicting case complexity, determined by F1 fidelity score (Fig. 1A). Models were sequentially executed using clinical factors, OPI metrics, or both. CatBoost models using both clinical and OPI covariates performed the best, with F1 score of 0.87, followed by CatBoost using clinical factors only with F1 score of 0.85 (Fig. 1B). CatBoost models with OPI metrics only performed the worst with an F1 score of 0.58 (Fig. 1B). Additionally, the performance of clinical and OPI covariates together did not appear to have a summative effect and in some instances, such as the Random Forest models, distracted away from the predictive power of clinical factors on hernia repair complexity (Fig. 1A and B). This is further illustrated by the covariates with the highest SHAP values, which indicate their average impact on the predictive model. These covariates are largely consistent between the clinical factors-only model and the combined clinical factors and OPI model, with only slight variations in their relative weights (Fig. 1C). Moreover, the combined clinical factor and OPI model have lower absolute SHAP values overall for each top ranked covariate, further underscoring the potential deleterious effect of OPIs on predictive modeling of case complexity overall (Fig. 1C).

### Predictive value of OPIs on case complexity does not improve after accounting for attending versus trainee primary surgeon

**Fig. 2:**
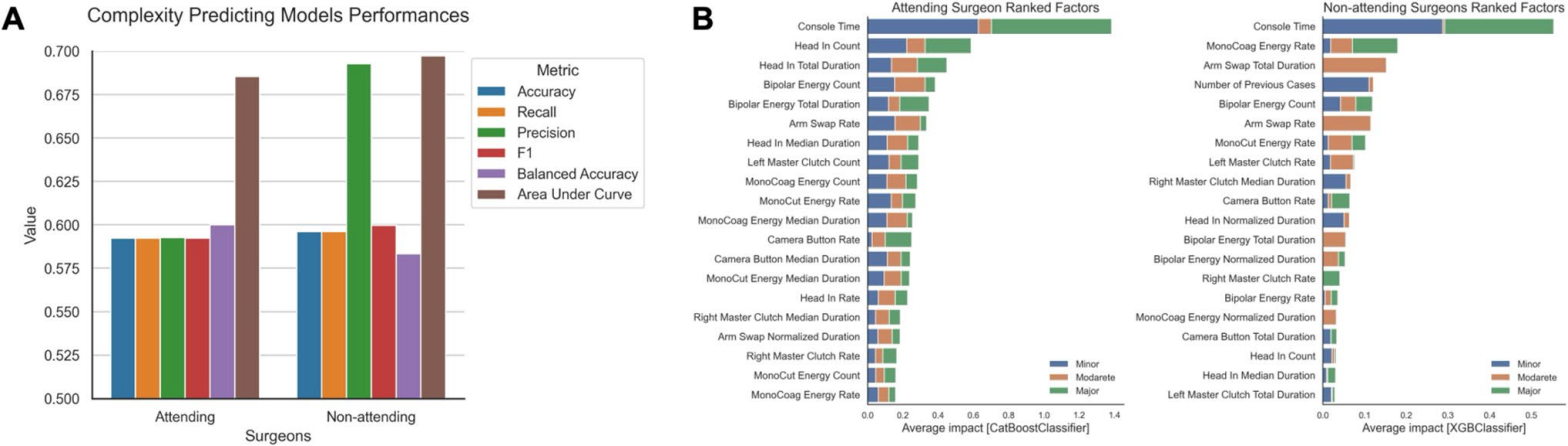
Performance of OPI factor-only machine learning models predicting case complexity after accounting for surgeon experience. **A** Performance metrics of best performing complexity predictive models using OPI factors only, grouped by attending versus non-attending primary surgeon. **B** SHAP values of top ranked factors in each model, segmented by prediction for minor, moderate, and major complexity cases

Recognizing that surgeon expertise could greatly influence operator behavior in face of intraoperative technical complexity, cases were subclassified into those performed by attending versus non-attending surgeons as primary surgeon (Fig. 2). Iterative machine learning models were employed using OPI covariates among each group and the best performing ensemble model was selected. OPIs did not predict case complexity with high fidelity in attending surgeon cases with minimal difference in F1 scores (F1 0.59), despite a slight but insignificant improvement in the non-attending surgeon model (F1 0.60; Fig. 2A). Covariates with the 20 highest SHAP values were ranked from each model accounting for non-attending versus attending surgeons (Fig. 2B). This revealed, despite varying SHAP values and thus absolute predictive weight, there is thematic conservation of OPIs related to head-in-console behavior, non-dominant hand clutch, and energy activation. This is concordant with the previous findings that found the same OPI metrics as the most predictive of postoperative complications [11].

### Dimensional reduction of OPIs captures evolution of surgeon skill from novice to expert in robotic ventral hernia repair

**Fig. 3:**
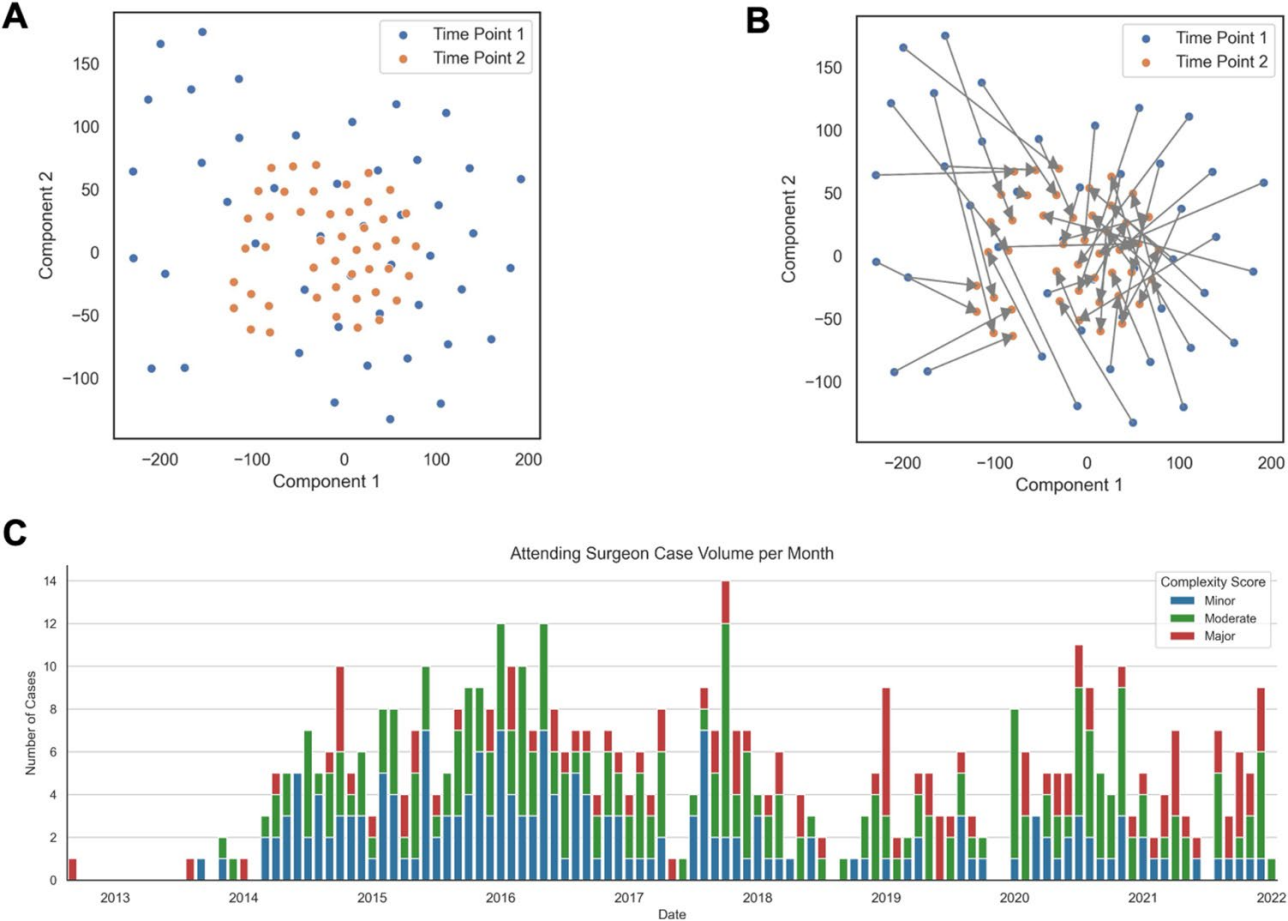
tSNE dimension reduction with Euclidean distances estimates evolution of OPI metrics over case and time along expert surgeon’s learning curve. **A** Representative scatterplots of first two consecutive timepoints by component after tSNE dimension reduction of OPI metrics. **B** Representative calculation of Euclidean distances of OPIs between first two consecutive timepoints. **C** Histogram depicting distribution of hernia cases with corresponding complexity across time, binned by one-month intervals along eight-year dataset

The above results led to the hypothesis that OPI profiles evolve over the course of a surgeon’s expertise level and that intraoperative complexity is a function of both clinical factors and surgeon skill acquisition. To demonstrate this, we performed t-distributed stochastic neighbor embedding dimensional reduction of OPIs and generated Euclidean distances for OPIs between successive cases to capture evolution of OPIs over case-time (Fig. 3A and B). Expert surgeon cases were binned by month for the entire dataset to track distribution of case complexity over time. This reveals that most low-complexity cases were in the earlier months as opposed to the latter half of the dataset were mainly comprised of medium and high-level complexity (Fig. 3C). OPI metrics after t-SNE dimensional reduction at each binned timepoint is further represented in an animated video in Supplemental Video 1.

**Fig. 4:**
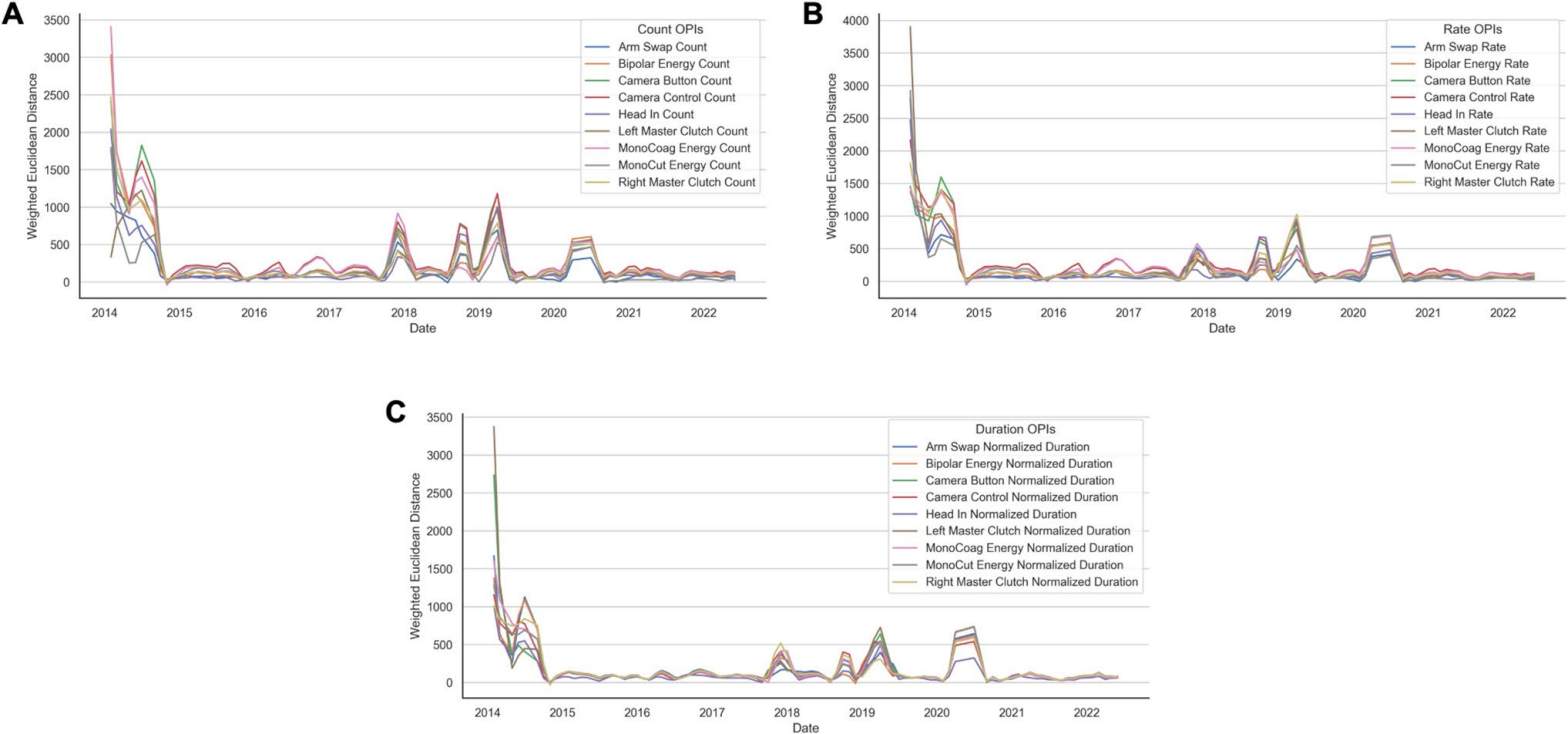
Learning curves generated for expert surgeon using weighted Euclidean distances of following grouped OPI metrics after tSNE dimension reduction with Savitzky–Golay filter, weighted for time: **A** OPIs by count, **B** OPIs by rate, and **C** OPIs by normalized duration

Supplemental Video 1 demonstrates timeline data of OPI Euclidean distances between successive cases with Savitzky–Golay filter and weighted by time, capturing a reflection of skill acquisition of the expert surgeon over time throughout the 8-year dataset. Each event-based OPI is presented as an absolute count, rate, and normalized duration (Fig. 4A–C, respectively). Euclidean distances for OPIs were much greater in the beginning of the surgeon’s cases with a rapid reduction by the first 10 months of the series. Following this, the absolute value and variance of distances remain tightly bound despite introduction of increasingly complex cases. There were a few upticks in subsequent years in the OPI Euclidean distance trends corresponding to evolution of surgeon herniorrhaphy technique, but absolute values of weighted distances do not reach peaks close to those reminiscent of the beginning 10 months of the surgeon’s robotic herniorrhaphy experience.

### Skill segmentation based on OPI-derived learning curves does not improve performance of OPI as independent predictors of case complexity

**Fig. 5:**
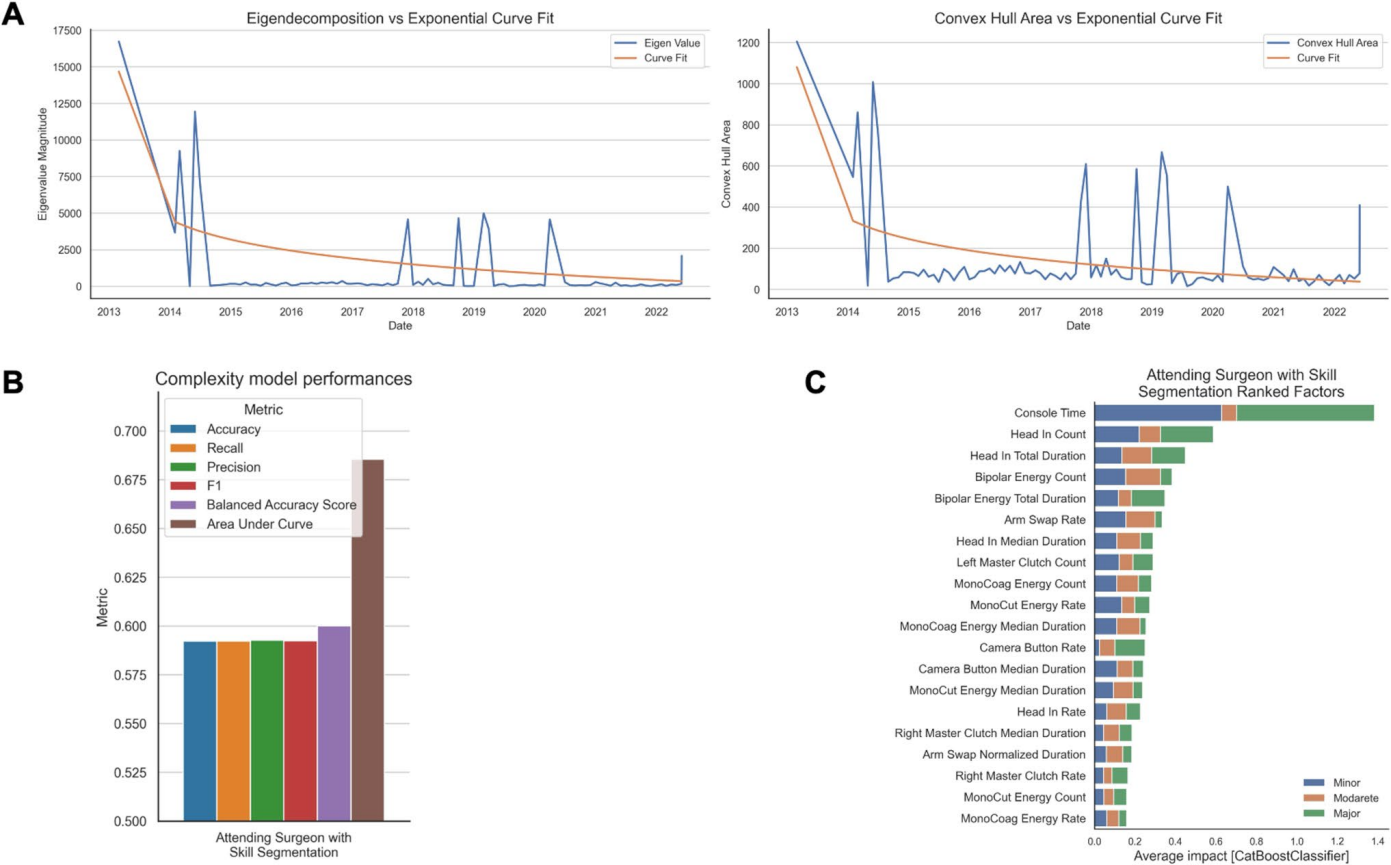
Sensitivity analyses after skill segmentation on attending surgeon cases. **A** Exponential decay fit on eigendecomposition and convex hull analyses on attending surgeon OPI Euclidean distances over time with segmentation between Novice and Expert surgeon phases (transition point 4/30/2017). **B** Performance data of best performing ensemble machine learning model predicting case complexity using OPI features only after skill segmentation. **C** SHAP values of top ranked factors in each model, segmented by prediction for minor, moderate, and major complexity cases

While Fig. 2 demonstrates insignificant differences in OPI predictive value on case complexity between attending and non-attending surgeons, we hypothesized that this may be affected by inherent limitations related to trainees having significantly shorter tenures at our academic center and lower consecutive case volume. This limitation would not capture how evolution of skill acquisition may affect operator behavior and subsequently OPIs’ abilities to predict case complexity. To address these limitations, eigendecomposition and convex hull analyses were used on the attending surgeon cases to capture temporal transformation of OPI Euclidean distances, followed by best fit exponential decay functions to estimate a trough level of when OPI matrices started to stably converge, representative of a transition from active skill acquisition (or “Novice” surgeon) to skill mastery (or “Expert” surgeon). Similar analyses were completed using convex hull to determine the transition point of when the smallest convex set of OPI Euclidean distances are detected in time. These results are highlighted in Fig. 5A and both demonstrated the same transition point of 4/30/2017 when the attending surgeon transition from Novice to Expert surgeon.

Using this case date as the transition point, cases were grouped a priori as Novice versus Expert surgeon cases and re-employed in our previously described iterative ensemble machine learning models. This sensitivity analysis revealed that skill segmentation did not significantly improve OPI ability to independently predict case complexity (F1 score 0.59; Fig. 5B). Consistent with previous figures, this analysis reveals that post-segmentation models continue to thematically conserve features related to head-in-console behavior, non-dominant hand clutch, and energy activation (Fig. 5C).

## Discussion

Analysis of robotic data metrics from 561 ventral hernia repairs revealed that OPIs alone do not effectively predict case complexity. However, these indicators become markedly more predictive when examined during the early phase of surgical training, as shown through sensitivity analyses of OPI metrics from trainee operations. By analyzing an 8-year learning curve spanning hernia repairs of varying complexity, we discovered an important interaction: while OPIs can indicate both case complexity and surgeon skill as previous studies suggest, the relationship between these factors is interdependent [17, 19, 29]. This interaction emerged through dimensional reduction analyses of OPI data across consecutive procedures.

The original hypothesis of this study sought to elucidate the independent predictive value of OPIs on case complexity during robotic ventral hernia repairs. The use of objective behavior-centric metrics would provide unbiased methods to define case complexity with significant implications in intraoperative decision making, perioperative patient care, and post hoc procedural reimbursement. Models using clinical factors only performed robustly in predicting case complexity, likely due to the fact that conventional definitions of hernia complexity use clinical and patient factors a priori. However, our results demonstrate that OPIs alone are poor predictors of case complexity in robotic ventral hernia repairs compared to patient clinical factors despite use of iterative ensemble machine learning models. Quality checks were additionally performed to confirm that our failure to reject our null hypothesis was not driven by significant biases related to highly correlative covariates using Pearson correlations between clinical and OPI covariates with case complexity. Moreover, the highest ranked factors contributing the most predictive weight in the clinical only and clinical + OPIs models are the same, emphasizing the non-additive effect of adding OPIs to complexity prediction models. This may potentially be related to how OPIs captured during the present study focus primarily on event-based OPIs rather than kinematic OPIs, which may provide greater insight into complexity prediction and certainly an avenue of future experiments. Our findings, however, elucidate a relative rank in predictive value among the canonical clinical predictive factors of hernia complexity, as indicated by SHAP values, imparting additional insight into a more granular definition of how to grade RVH case complexity clinically.

The OPI-derived learning curve is derived using a novel approach combining case complexity along with multidimension reduction and weighted Euclidean distances in operator metrics over case and time. The scattered dispersion of OPI metrics and wide Euclidean distances between cases in the beginning of this curve can be reflective of coarse technique. The distances start to narrow and metrics become tighter as the steep learning process is completed. Throughout the evolution of this surgeon’s hernia practice, several modifications took place between 2017 and 2021, including incorporation of new approaches and eventual transition from a largely intraperitoneal robotic-assisted approach to an almost exclusively totally extraperitoneal approach. With increasing case complexity, the employed techniques broadened to include not only intraperitoneal onlay repairs but also minimally invasive complex abdominal wall reconstructions such as robotic-assisted laparoscopic bilateral transversus abdominis releases. The learning curves highlight how introduction of new techniques and evolution of practice are represented by minor peaks in Euclidean distances that are far lower than those found in the beginning of the curve at novice level. This suggests mastery of the robotic platform confers durable skill retention and ease of integrating new surgical approaches in complex hernia repairs. Chronologic ordering of hernia operations in our dataset further reveals a temporal relationship behind case complexity and increasing surgeon case volume, potentially further obscuring the ability of OPIs to independently predict case complexity in isolation. This pattern aligns with established learning theory: as the surgeon moves along a learning curve, they improve surgical technique, represented by decreasing and minimal variation in operative metrics despite increased complexity of case blend. While selection bias may exist, this is mitigated by analysis of consecutive robotic hernia repairs, irrespective of patient or hernia features. The relative stability of OPI dispersion on component analysis after t-SNE dimension reduction despite increased complexity in operative cases demonstrates how operative metrics become less relevant in informing about case complexity with increasing surgeon expertise.

Our study reveals that OPIs, while novel, are not effective independent predictors of case complexity. We investigated this by comparing cases with attending versus non-attending primary surgeons and by applying eigendecomposition and convex hull segmentation to our attending surgeon OPI-derived learning curves. These investigations stemmed from our hypothesis that surgical performance results from the interaction between clinical expertise and case complexity. Unsurprisingly, the most accurate complexity prediction models incorporated clinical features, as the accepted definition of complexity is primarily based on clinical criteria established through Delphi consensus. Notably, our iterative machine learning models using either clinical features alone or clinical features combined with OPIs consistently outperformed conventional logistic regression models, validating our methodology. Our results identify a hierarchy of feature importance within the Delphi consensus/Ventral Hernia Working Group criteria for predicting complexity, potentially offering new insights into the clinical definition of hernia complexity.

Limitations of this study are principally related to how operative cases examined are derived from a single-expert robotic surgeon’s experience. While this limits the generalizability of our findings to all robotic surgeons, it allows for a robust longitudinal series over eight years of experience during which the surgeon has progressed from a novice to master robotic hernia surgeon. Along this vein, the specific highly ranked OPI covariates predicting case complexity would not necessarily be the same for a similar series of robotic herniorrhaphy from another surgeon. As robotic technology evolves, the specific OPIs highlighted in this study may change in relevance. Nevertheless, our novel methodology demonstrates that longitudinal OPI metrics can capture the interplay between case complexity and surgeon experience, potentially serving as objective measures of skill acquisition. Future related experiments would include OPI capture combined with operative segmentation during robotic hernia repair, particularly for portions with complete trainee autonomy, and multi-institutional validation. This enables for meaningful derivation of OPI-based learning curves for trainees and comparison of those trends with the longitudinal curves generated from the expert surgeon’s 8-year experience.

## Supplementary Information

Below is the link to the electronic supplementary material.

Supplementary file1 (MOV 8244 KB)—Animated representation of successive OPI metrics after tSNE dimension reduction used to calculated weighted Euclidean distances for eventual learning curve. In the PDF version of this article, please click anywhere on the figure or caption to play the video in a separate window.
